# Identification of Neutralizing Monoclonal Antibodies Targeting Novel Conformational Epitopes of the Porcine Epidemic Diarrhoea Virus Spike Protein

**DOI:** 10.1038/s41598-019-39844-5

**Published:** 2019-02-21

**Authors:** Chia-Yu Chang, Ivan-Chen Cheng, Yen-Chen Chang, Pei-Shiue Tsai, Seiu-Yu Lai, Yu-Liang Huang, Chian-Ren Jeng, Victor Fei Pang, Hui-Wen Chang

**Affiliations:** 1 Graduate Institute of Molecular and Comparative Pathobiology, School of Veterinary Medicine, National Taiwan University, Taipei, 106 Taiwan; 2 School of Veterinary Medicine, National Taiwan University, Taipei, 106 Taiwan; 3 Animal Health Research Institute, Council of Agriculture, New Taipei City, 251 Taiwan

## Abstract

Since 2010, newly identified variants of porcine epidemic diarrhoea virus (PEDV) have caused high mortality in neonatal piglets which has devastated the swine industry. The spike (S) glycoprotein of PEDV contains multiple neutralizing epitopes and is a major target for PEDV neutralization and vaccine development. To understand the antigenicity of the new PEDV variant, we characterized the neutralizing epitopes of a new genotype 2b PEDV isolate from Taiwan, PEDV Pintung 52 (PEDV-PT), by the generation of neutralizing monoclonal antibodies (NmAbs). Two NmAbs, P4B-1, and E10E-1–10 that recognized the ectodomain of the full-length recombinant PEDV S protein and exhibited neutralizing ability against the PEDV-PT virus were selected. Recombinant truncated S proteins were used to identify the target sequences for the NmAbs and P4B-1 was shown to recognize the C-terminus of CO-26K equivalent epitope (COE) at amino acids (a.a.) 575–639 of the PEDV S. Interestingly, E10E-1–10 could recognize a novel neutralizing epitope at a.a. 435–485 within the S1^A^ domain of the PEDV S protein, whose importance and function are yet to be determined. Moreover, both NmAbs could not bind to linearized S proteins, indicating that only conformational epitopes are recognized. This data could improve our understanding of the antigenic structures of the PEDV S protein and facilitate future development of novel epitope-based vaccines.

## Introduction

Porcine epidemic diarrhoea virus (PEDV) causes porcine epidemic diarrhoea (PED), a highly contagious disease characterized by acute watery diarrhoea, vomiting, and dehydration, and with a high mortality rate particularly in suckling piglets^1,2^. The first outbreak of PED was recorded in the European and Asian swine industries in the early 1970s and then spread to many countries^3,4^. In 2010, novel and highly virulent PEDV strains were identified in China, which later spread to several countries^1,5,6^. These new variants of PEDV have caused high morbidity and mortality in neonatal piglets, resulting in serious economic loss to the swine industry^1,7^. Thus, there is an urgent need for in-depth and comprehensive studies on the antigenicity and immunogenicity of PEDVs in order to facilitate disease control and eradication.

PEDV is a single-stranded RNA virus, approximately 28 kb in size, which belongs to the genus *Alphacoronavirus*^8^. The PEDV genome comprises of seven open reading frames (ORFs) that encode for non-structural proteins responsible for viral genome replication and transcription, as well as four structural proteins, namely spike (S), envelope (E), membrane (M), and nucleocapsid (N) proteins^8,9^. Among the structural proteins, the S glycoprotein plays a critical role in host affinity, virus-cell recognition, membrane fusion and entry^1,10,11^. Moreover, it contains multiple neutralizing epitopes that make it a suitable target for vaccines designed to neutralize PEDVs^12–14^.

Several neutralizing epitopes have been identified on the S protein of the PEDVs such as the CO-26K equivalent epitope (COE epitope)^12^, the antigen epitope motif recognized by the monoclonal antibody (mAb) 2C10 at the C-terminal end of the S protein^13^, and a neutralizing epitope named S1D^14,15^. Recently, five core cell attachment domains of the PEDV S1 protein, S1^0^, S1^A^, S1^B^, S1^C^, and S1^D^, were proposed based on the three-dimensional structures cryo-EM of *Alphacoronaviruse*s^16,17^. The proposed S1^0^ domain has a sialic acid binding domain^10^. The S1^B^ domain is a common location for receptor binding in coronaviruses and can be a potential neutralizing target for the new PEDV variant capable of eliciting the production of cross-neutralizing antibodies against different PEDV strains^17^. Furthermore, several linear B-cell epitopes of PEDV overlapping with S1D have been shown to neutralize both the historic strain as well as the new variant PEDV strains^18^. Till date, no neutralizing epitope has been identified in the S1^A^ domain of the historic or the new PEDV variant strains.

To understand the antigenicity of PEDV, we generated a panel of neutralizing mAbs (NmAbs) by immunizing BALB/c mice with inactivated PEDV viral particles. The neutralizing epitopes of the PEDV-PT S protein, recognized by two of the NmAbs, were characterized using a series of C-terminal truncated recombinant PEDV-PT S proteins under native and denaturing conditions. This approach allows us to identify conformational epitopes of PEDV-PT strain and helps in understanding the antigenicity and pathogenesis of the G2b PEDV.

## Results

### Generation and purification of anti-PEDV spike mAbs

To map the epitopes of the new variants of PEDV, the hybridomas originating from PEDV-immunized mice were generated that secreted mAbs into the culture supernatant. These mAbs were collected from the supernatant and were used for immunocytochemical (ICC) staining with the full-length PEDV spike-expressing human embryonic kidney (HEK) 293 cells. Three mAbs, P4B-1, E10E-1–10, and R7H-2, which had a binding titre greater than 1:160 for PEDV spike-expressing HEK293 cells, were selected and purified. One mAb, UNK-1, which had no binding affinity for the PEDV spike-expressing HEK293 cells, was also selected to be a negative control for the subsequent assays. After purification of the mAbs along with the protein L, which targets the Fc region of the mice-originated antibodies, the pure mAbs of P4B-1, E10E-1–10, R7H-2, and UNK-1 were obtained at 400 μg/mL, 250 μg/mL, 900 μg/mL, and 400 μg/mL, respectively.

### Determination of neutralizing antibodies

After purification and quantification of mAbs, the neutralizing ability of each mAb against the G2b Taiwan PEDV-PT strain was assessed using the virus neutralizing assay. The starting concentration for each mAb in the neutralizing assay was 20 μg/mL, with a two-fold serial dilution to obtain a final neutralizing concentration of 2.5 μg/mL. The mAbs, P4B-1, and E10E-1–10, possessed potent neutralizing ability that completely blocked the CPE of the Taiwan PEDV-PT strain under 5 μg/mL. On the contrary, the mAbs, R7H-2, and UNK-1, showed no neutralization ability against PEDV-PT even at 20 μg/mL.

### Generation of various truncated forms of the Taiwan PEDV-PT spike protein

**Figure 1:**
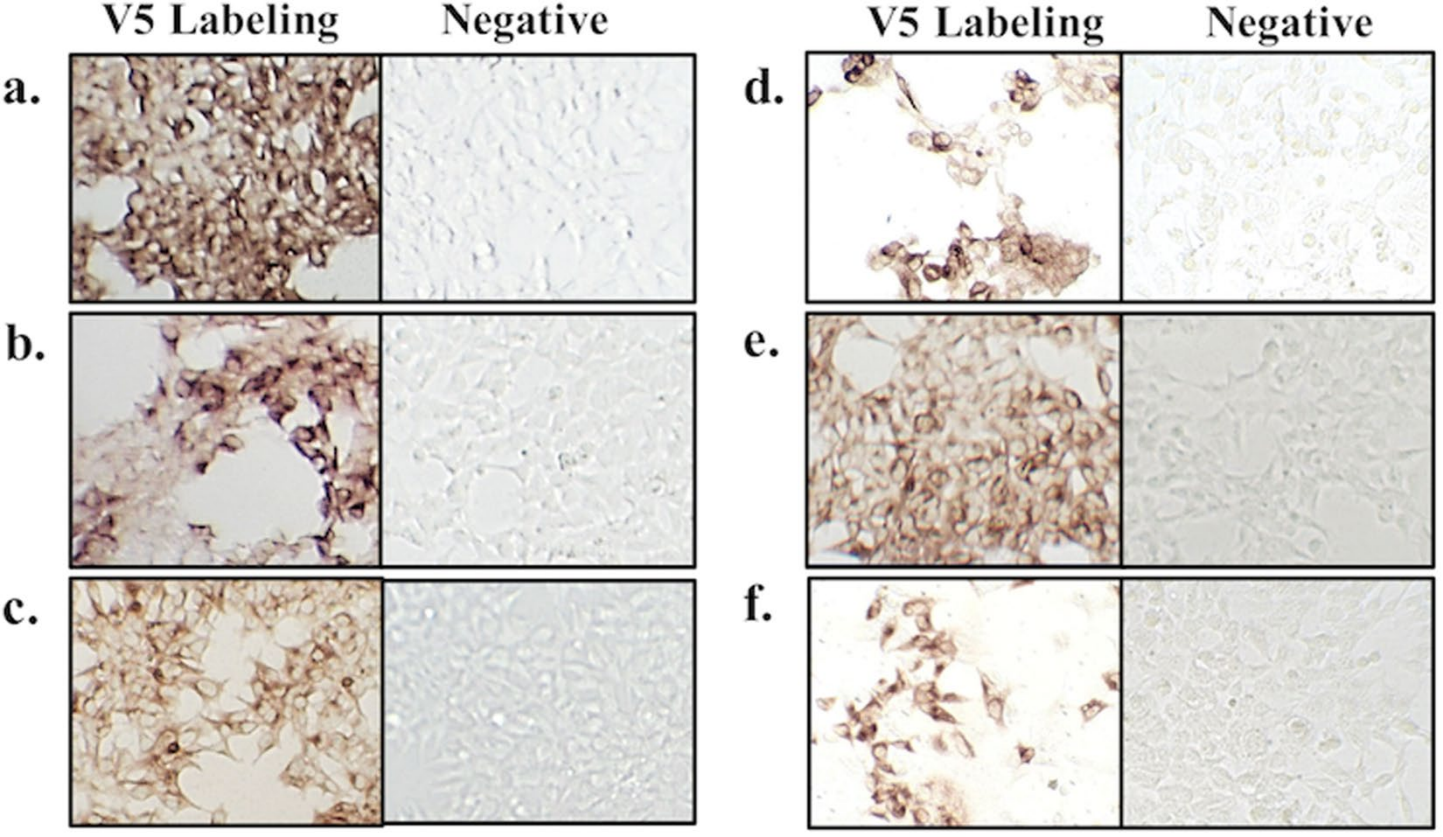
Immunocytochemical (ICC) staining of HEK293 cells expressing various truncated spike protein of PEDV-PT. Six truncated spike proteins-expressing HEK293 cells with a V5 tag were constructed and the construction efficiency was estimated by the specific binding of the anti-V5 tag antibody to these HEK cells. The signals were detected using the polyclonal anti-rabbit/mouse immunoglobulin EnVision-DAB+ system and only the secondary antibody was used to stain the negative controls to exclude nonspecific signals. The staining profile corresponding to truncations of the spike protein are as (**a**) a.a. 1–435; (**b**) a.a. 1–485; (**c**) a.a. 1–501; (**d**) a.a. 1–509; (**e**) a.a. 1–575; (**f**) a.a. 1–639.

**Figure 2:**
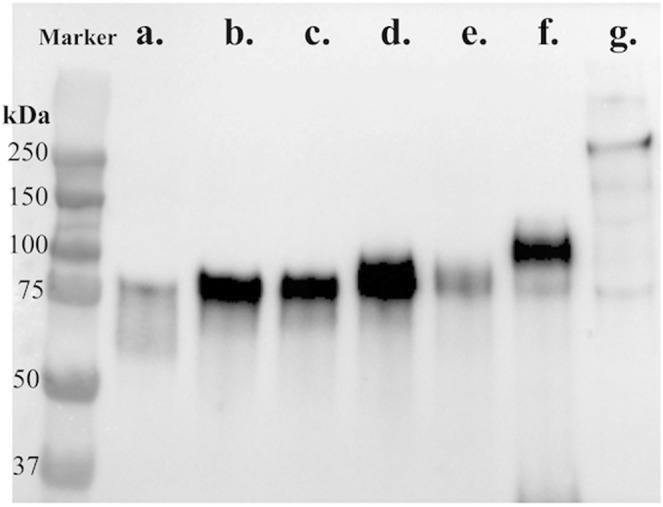
Western blotting of HEK293 cells expressing various truncated spike proteins. Cell lysates of HEK293 cells expressing different lengths of S protein were separated by SDS-PAGE, transferred to PVDF membranes, probed with an anti-V5 tag antibody, and detected using HRP-conjugated secondary goat anti-mouse immunoglobulin antibody. The protein ladder/marker is shown in kilodalton (kDa). The bands corresponding to the various truncations of the spike protein are (**a**) a.a. 1–435; (**b**) a.a. 1–485; (**c**) a.a. 1–501; (**d**) a.a. 1–509; (**e**) a.a. 1–575; (**f**) a.a. 1–639; (**g**) full-length spike protein.

A commercially synthesized codon-optimized complete gene sequence of the PEDV-Pintung 52 S protein (PEDV-PT; GenBank accession No. KP276252) was used to amplify DNA amplicons of varying lengths that encode different truncated forms of the S protein genes. Using the primer pairs listed in Supplementary Table 1, DNA sequences of different lengths were amplified namely S^1-435^ (1305 bp), S^1-485^ (1455 bp), S^1-501^ (1503 bp), S^1-509^ (1527 bp), S^1-575^ (1725 bp), S^1-639^ (1917 bp) and the full-length S ectodomain (4086 bp) of the PEDV-PT strain. These DNA fragments were digested with restriction enzymes and ligated into the pcDNA3.1-V5-His vector. Seven plasmids containing the V5-tag, namely pcDNA3.1-PEDV S^1-435^-V5-His, pcDNA3.1-PEDV S^1-485^-V5-His, pcDNA3.1-PEDV S^1-501^-V5-His, pcDNA3.1-PEDV S^1-509^-V5-His, pcDNA3.1-PEDV S^1-575^-V5-His, pcDNA3.1-PEDV S^1-639^-V5-His, and pcDNA3.1-PEDV S-V5-His were constructed. We have shown the construction of the full-length spike ectodomain of the Taiwan PEDV-PT strain in a previous publication^19^. The expression levels of various truncated recombinant S proteins, S^1-435^, S^1-485^, S^1-501^, S^1-509^, S^1-575^, and S^1-639^ of the PEDV-PT strain, were successfully detected using ICC staining with the anti-V5 tag antibody (Fig. 1), as well as western blotting (Fig. 2). The V5 tag-positive HEK293 cells for each construct occupied 70% to 90% of the total population. The positive ratio of each construct was adjusted between 60% and 70% by mixing with untransfected HEK293 cells, which were used as internal negative control in the ICC staining. The corresponding molecular weights of S^1-435^, S^1-485^, S^1-501^, S^1-509^, S^1-575^, S^1-639^, and the full-length spike protein were detected to be approximately 60, 75, 75, 80, 85, 90, and 250 kDa, respectively (Fig. 2).

### Mapping the epitopes of the PEDV-PT by ICC staining

**Figure 3:**
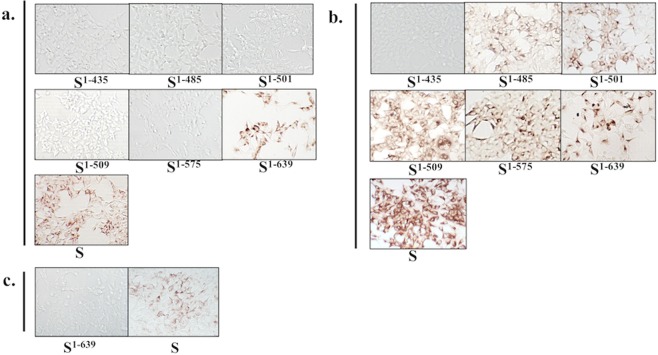
Mapping epitopes of neutralizing mAbs (P4B-1 and E10E-1–10) and the non-neutralizing mAb (R7H-2) by immunocytochemical (ICC) staining of different truncated spike proteins of PEDV-PT. The HEK293 cells expressing the various truncated spike proteins were fixed with 80% acetone and probed with mAbs, P4B-1, E10E-1–10, and R7H-2, respectively and The specific binding of the antibodies was detected using the polyclonal anti-rabbit/mouse immunoglobulin EnVision-DAB+ system. The staining of the cells expressing various constructs are shown as follows (the superscripts denote the specific amino acid sequence for the truncation): S^1-435^, S^1-485^, S^1-501^, S^1-509^, S^1-575^, S^1-639^ and full-length S. The six different truncated spike proteins and full-length spike (S) were stained with (**a**) P4B-1 and (**b**) E10E-1–10 while the S^1-639^ truncated spike protein and full-length spike (S) were stained with (**c**) R7H-2.

As summarized in Table 1, the two NmAbs, P4B-1 and E10E-1–10 and the non-neutralizing mAb, R7H-2, all exhibited the ability to recognize full-length PEDV spike-expressing HEK293 cells but were unable to recognize PEDV S^1-435^-expressing HEK293 cells in ICC staining. More precisely, P4B-1 recognized PEDV S^1-639^ expressing HEK293 cells but did not recognize the HEK293 cells expressing S^1-575^, S^1-509^, S^1-501^, S^1-485^, S^1-435^ of PEDV-PT (Fig. 3). In other words, P4B-1 was unable to bind to the amino acids upstream of a.a. 575 of the spike protein of PEDV-PT. Therefore, the targeting epitope of P4B-1 must be located between a.a. 575–639 on the PEDV spike protein. Surprisingly, E10E-1–10 exhibited good binding affinity for HEK293 cells expressing PEDV proteins S^1-639^, S^1-575^, S^1-509^, S^1-501^, and S^1-485^, but with reduced binding affinity for the PEDV S^1-435^. This evidence obtained from the ICC staining confirmed this NmAb recognizing the novel epitope present within the 435–485 a.a. region of PEDV-PT spike protein (Fig. 3). In contrast, the non-neutralizing mAb, R7H-2, that recognized only the full-length PEDV spike-expressing HEK293 cells, was unable to bind to the PEDV spike protein upstream of a.a. 639 (Fig. 3). These results suggest that the targeting epitope of R7H-2 is downstream of a.a. 639 of the PEDV spike protein.

**Table 1:** A summary of the affinity-binding results of different mAbs to various truncated S proteins by immunocytochemical (ICC) staining.

mAb	S^1-435^	S^1-485^	S^1-501^	S^1-509^	S^1-575^	S^1-639^	S
P4B-1	−	−	−	−	−	+	+
E10E-1–10	−	+	+	+	+	+	+
R7H-2	NE	NE	NE	NE	NE	−	+

The ICC results are shown as positive (+), negative (−) and non-examined (NE). S^1-435^, S^1-485^, S^1-501^, S^1-509^, S^1-575^, S^1-639^ and S: Cells expressing amino acid a.a. 1–435, 1–485, 1–501, 1–509, 1–575, 1–639 and full-length spike protein.

### ELISA results showing reactivity of mAbs to various truncated PEDV S proteins

**Figure 4:**
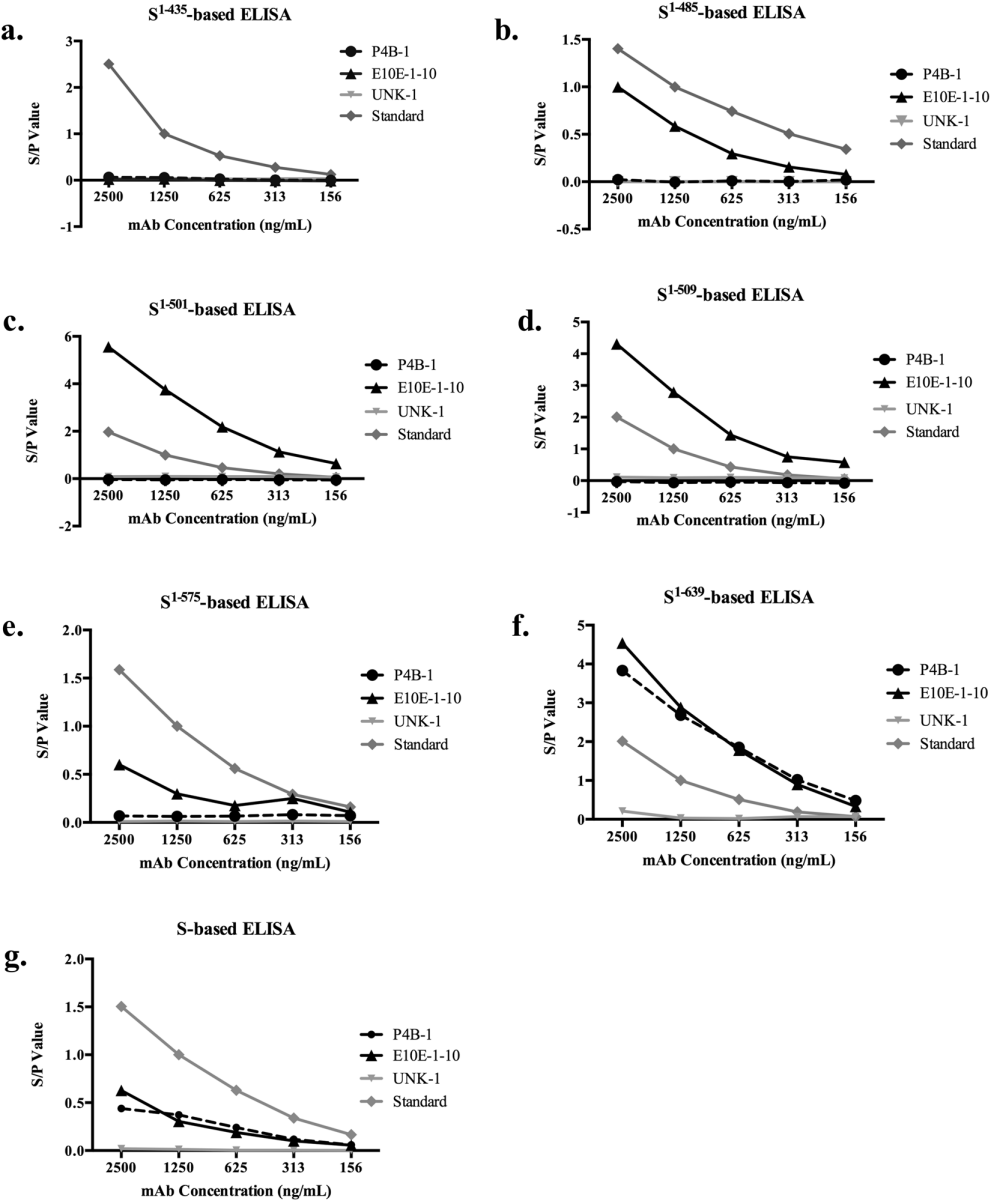
Mapping epitopes by ELISA targeting different truncated PEDV S proteins. The six truncated as well as full-length spike protein of PEDV-PT were coated into separate wells and probed with two-fold serially diluted mAbs, P4B-1 and E10E-1–10. The non-PEDV recognizing mAb, UNK-1, was added as the external control for ELISA under the same dilution conditions. The two-fold serially diluted anti-V5 tag antibody was used as the standard to show the dilution effect of the binding affinity assay. The data is represented as the S/P ratio. The wells incubated with only secondary antibodies and the wells incubated with anti-V5 tag antibody at 1,250 ng/mL were used as negative and positive controls for S/P ratio respectively. The results of the ELISA for the various truncations of the spike proteins are shown as follows: (**a**) S^1-435^; (**b**) S^1-485^; (**c**) S^1-501^; (**d**) S^1-509^; (**e**) S^1-575^; (**f**) S^1-639^ and (**g**) full-length S.

To further confirm the reactivity of each mAb to the targeting epitopes, ELISAs were performed using plates coated with various truncated PEDV spike proteins. The PEDV NmAbs (P4B-1 and E10E-1–10) and the non-PEDV recognizing mAb (UNK-1) were all serially diluted to conduct the ELISA. As shown in Fig. 4, P4B-1 had a binding affinity with appropriate dilution effects toward the purified full-length PEDV spike protein, as well as PEDV S^1-639^, but had its affinity towards purified PEDV proteins, S^1-575^, S^1-509^, S^1-501^, S^1-485^ and S^1-435^ was weak. However, E10E-1–10, which targets a novel neutralizing epitope as shown with ICC staining, was also capable of binding to the full-length PEDV spike protein as well as truncated PEDV proteins, S^1-639^, S^1-575^, S^1-509^, S^1-501^, and S^1-485^ with appropriate dilution effects. Similar to the results obtained by ICC staining, E10E-1–10 showed no binding ability toward PEDV S^1-435^ by ELISA and therefore, an NmAb targeting a novel epitope of the new variant of PEDV specifically at a.a. 435–485 in the S1 region was confirmed. The non-PEDV recognizing mAb, UNK-1 showed no binding affinity towards the various truncations of the spike protein and hence, it was used as the external control for the ELISA assay.

### Binding affinity of NmAbs to linearized epitopes

**Figure 5:**
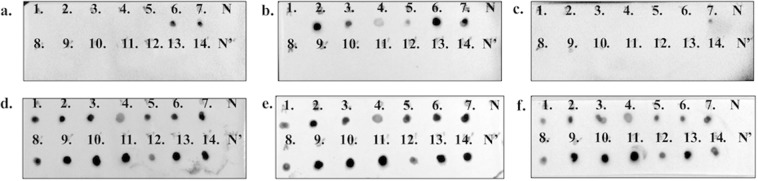
Linear epitope mapping by immunodot blotting with native and denatured proteins. The conformational proteins of six truncations as well as full-length spike of PEDV-PT were directly dotted on the first row of each membrane in the order of protein length. After denaturing at 95 °C in the reducing agent, the proteins were dotted on the second row of each membrane. The dot-blot profiles of the conformation proteins for the six truncations, S^1-435^, S^1-485^, S^1-501^, S^1-509^, S^1-575^, S^1-639^ and full-length S are shown in panels 1–7 while the profiles for the denatured S^1-435^, S^1-485^, S^1-501^, S^1-509^, S^1-575^, S^1-639^ and full-length S proteins are in panels 8–14, respectively. N: negative control without the denaturing process; N’: negative control with the denaturing process. (**a**–**c**) The membrane stained with P4B-1, E10E-1–10, and R7H-2, respectively. (**d**–**f**) The stripped membrane re-probed with anti-V5 tag antibody.

To further determine whether the binding of neutralizing epitopes require conformational integrity, immunodot blotting was performed by denaturing the various truncated PEDV spike proteins. The conformational proteins as well as the denatured proteins of S^1-435^, S^1-485^, S^1-501^, S^1-509^, S^1-575^, S^1-639^, and the full-length spike of PEDV-PT were dotted onto the membrane. As shown in Fig. 5(a–c), P4B-1 was able to detect the conformational S^1-639^ and the full-length spike protein on the membrane but was unable to detect the proteins upstream of a.a. 575 of spike, consistent with the findings from the ELISA and ICC staining. The other NmAb, E10E-1–10, could bind to S^1-485^, S^1-501^, S^1-509^, S^1-575^, S^1-639^ and the full-length spike proteins in conformational structures. The R7H-2, a spike-recognizing but non-neutralizing mAb, was only capable of binding to the conformational full-length spike protein. P4B-1, E10E-1–10, and R7H-2 were unable to stain the denatured target proteins on the membrane, indicating the loss of binding affinity after protein linearization. Moreover, to ensure all the proteins were equally dotted on the membrane, the results of mAb-stripping and re-probing with anti-V5 tag antibodies are shown in Fig. 5(d–f).

### Modelling of PEDV S protein

**Figure 6:**
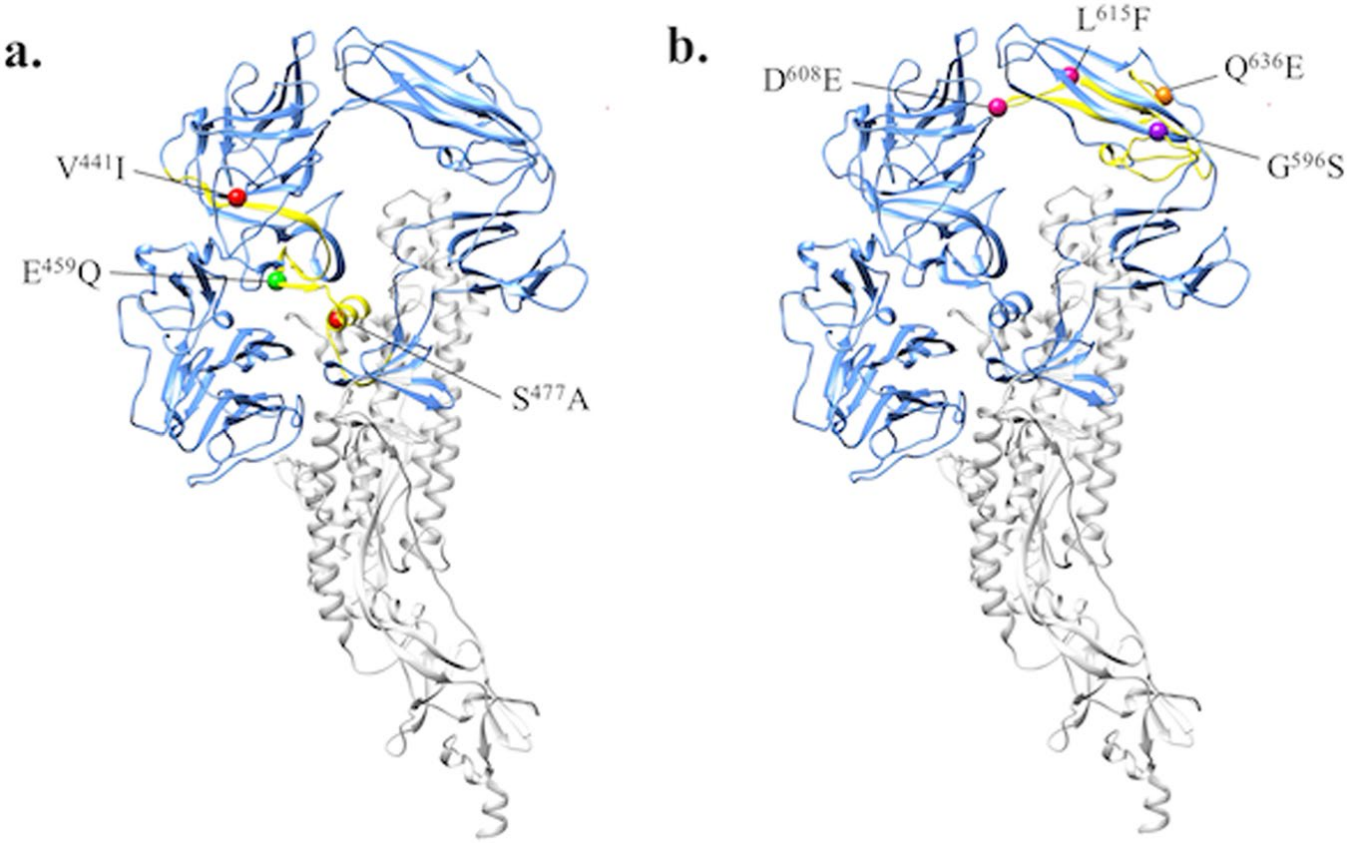
Molecular modelling of the recombinant spike (S) ectodomain protein. The recombinant ectodomain of S protein of PEDV-PT strain was modelled using SWISS model and edited using UCSF ChimeraX. The S protein was present as monomer and the S1 and S2 regions of PEDV were displayed in light-blue and grey, respectively. The conformational neutralizing epitope regions characterized by the mAbs are given as follows: (**a**) E10E-1–10 mAb labelled in yellow; two mutations (V^441^I and S^477^A) on the epitope different from the CV777 strain were highlighted in red sphere and one mutation (E^459^Q) on the epitope different from the Taiwan historic PEDV strain (HC070225-S) was highlighted in green sphere. (**b**) P4B-1 mAb was labelled in yellow; the G^596^S mutation on the epitope different from the CV777 strain was highlighted in a purple sphere; mutations, D^608^E and L^615^F, on the epitope different from the Taiwan historic PEDV strain (HC070225-S) were highlighted in pink sphere. The orange sphere represented the common mutation (Q^636^E) noted on the epitope of PEDV-PT S protein that was different from both of CV777 and Taiwan historic PEDV (HC070225-S) strains.

A molecular model of recombinant PEDV S ectodomain protein was established using SWISS Model and re-edited using UCSF ChimeraX molecular modelling system. As shown in Fig. 6, the monomer of PEDV S protein was divided into S1 (a.a. 1–735, in light-blue) and S2 (a.a. 736–1378, in grey). The novel neutralizing epitope (a.a. 435–485 of the S protein), characterized by the E10E-1–10 NmAb labelled in yellow in Fig. 6(a), was inferred to be at the C terminal region of the S1^A^ domain. The two mutations, V^441^I, and S^477^A that were different from the CV777 strain were highlighted in red sphere; and the one mutation, E^459^Q that was different from the Taiwan historic PEDV HC070225-S strain was highlighted with a green sphere. On the other hand, the region of the neutralizing epitope (a.a. 575–639 of the S protein; labelled in yellow) characterized by the P4B-1 NmAb was deduced at the C-terminal region of the S1^B^ domain in Fig. 6(b). The a.a. mutation (G^596^S) different from the CV777 strain was highlighted with a purple sphere; and the a.a. mutations, D^608^E and L^615^F, different from the Taiwan historic PEDV strain HC070225-S were highlighted with pink spheres. The mutation, Q^636^E that was different from both CV777 and Taiwan historic PEDV strain HC070225 was highlighted with an orange sphere.

## Discussion

Since 2010, outbreaks of infections caused by new PEDV variants have been correlated to viral mutations in critical neutralizing epitopes of the spike protein^8,20^. Although several sequences of the new variant PEDV spike genes have been decoded^6,21^ and multiple domain architectures of the PEDV S protein have been predicted by three-dimensional structures of alphacoronaviruses^16^, the effects of genetic mutations on the antigenicity of PEDV remains poorly understood. A thorough evaluation of the neutralizing epitopes and antigenicity of the new PEDV variants is therefore needed. In the present study, we generated a panel of mAbs using PEDV-PT viral particles. By selecting mAbs that exhibited high binding affinity to full-length spike ectodomain-expressing HEK293 cells, we successfully identified two representative NmAb against PEDV-PT. The targeting epitopes identified by these two NmAbs were located at a.a. 575–639 and a.a. 435–485 of the PEDV spike protein, respectively, as shown by ICC staining and ELISA. Further, none of these NmAbs were able to bind to the linearized forms of the truncated spike proteins, indicating that they recognize the integral epitopes with appropriate conformation. Taken together, we discovered a novel conformational neutralizing epitope in the S1^A^ domain at a.a. 435–485, whose function has not been clearly determined in coronaviruses. In addition, we also discovered a conformational neutralizing epitope close to the C-terminus of the COE domain, at a.a. 575–639.

The neutralizing epitopes of the historic strains of PEDV on the S protein that have been previously published include the CO-26K equivalent epitope (COE epitope; a.a. 499–638 in the Brl strain; a.a. 501–640 in the PEDV-PT strain)^12^, 2C10 epitope (a.a. 1368–1374 in the CV777 strain; a.a. 1373–1379 in the PEDV-PT strain)^13^, and S1D (a.a. 636–789 in the CV777 strain; a.a. 640–794 in the PEDV-PT strain)^14^. In case of the new variants of PEDV, the domain B (a.a. 510–640 in the PEDV GDU and the PEDV-PT strain) and domain 0 in the S1 region (a.a. 1–219 in the PEDV GDU and the PEDV-PT strain) are demonstrated to have neutralizing epitopes^17^. Furthermore, Okda *et al*. also discovered several linear epitopes at the N-terminus of S2^18^. Previously, the identification of neutralizing epitopes of the historic PEDVs led to the proposal that several single nucleotide polymorphisms (SNPs), such as three serine amino acid substitutions in the COE epitope and two serine amino acid substitutions in the S1D epitope, may be related to newly-occurring global outbreaks^6,22^. In the present study, the NmAb, P4B-1, was able to recognize the epitope within a.a. 575–639 on the S protein, which overlaps with the C-terminus of the COE epitope, echoing the findings of previous studies^12^. As the S1^B^ domain consists of the main COE neutralizing epitope of PEDV and is the receptor binding domain for the majority of the coronaviruses, its importance has been emphasized in many studies^11,23,24^. Li *et al*. have demonstrated that the binding affinity of neutralizing antibodies could be dramatically altered by exchanging one amino acid on the S protein of PEDV^17^. Hence, mutations in S1^B^ domain, especially in neutralizing epitopes of the PEDV S protein, are speculated to represent the evolution of viral escape from antibodies, like the one evoked by CV777-based vaccine immunization of a historic PEDV strain infection^4,25^. Interestingly, the other NmAb, E10E-1–10, presented a high binding affinity toward a.a. 435–485 of the S protein that precludes the COE epitope and locates in the S1^A^ domain has also been identified in our study. To our knowledge, the function of the S1^A^ domain of the PEDV S protein has not been well studied. The S1^A^ domain of human coronavirus HCoV-NL63 is speculated to interact with heparan sulfate proteoglycans on the host cells to mediate the virus anchoring and infection^26^. The N-terminus of S1^A^ domain of bovine coronavirus has been shown to have two sugar binding loops which can recognize the carbohydrate receptor on the host cells^27^. Moreover, heparan sulfate has also been demonstrated as an attachment factor for PEDV^28^. However, as the actual topography of the PEDV trimeric S protein remains to be solved and the function of S1^A^ domain is yet to be determined, the actual interaction of the S1^A^ domain targeting mAb with these sugars or the heparan sulfate proteoglycans-binding domains on the PEDV S glycoprotein remains to be further evaluated. Visualization of the topology and interaction of PEDV S glycoprotein with these neutralizing antibodies for guiding future immunogen and therapeutics design is an important future goal.

Epitopes for antibody recognition can generally be divided into two main classes: linear and conformational forms. Linear epitopes are formed by a continuous sequence of amino acid in a protein, while conformational epitopes consist of amino acid sequences that are discontinuous in the protein but are brought together upon three-dimensional protein folding. It has been demonstrated that 90% of B cell-recognizing epitopes are conformational epitopes that result from the antigen internalizing process and special antigen-recognizing ability^29,30^. Many conformational epitope-targeting antibodies, including neutralizing antibodies, are particularly difficult to determine because the antibody-antigen complex is formed solely in the native structure of the protein. Over the past several decades, with the use of phage-display peptide probing, the neutralizing epitope of CV777, named 2C10 has been identified^13^. Using ELISA or Pepscan assays, several linear neutralizing epitopes have been discovered on the S2 glycoprotein subunit of new PEDV variants^18^. Additionally, two B cell epitopes, named SS2 and SS6, in the region of the S1D neutralizing epitope were evaluated using a combination of phage-display peptide probing and Pepscan^14,15^. However, these epitope mapping approaches focus mainly on linear epitopes^31^. Using ICC staining, ELISA, and denatured immunodot blotting, all of the B cell-recognizing epitopes identified in the present study were found to be conformational epitopes. Thus, these affinity-binding assays could serve as valuable platforms for studying the conformational epitopes of NmAbs. In conclusion, the NmAbs and various truncated S proteins constructed generated in the present study could contribute to a better understanding of the antigenicity and immunogenicity of highly virulent PEDVs, especially in revealing the antigenic role of S1^A^ domain, and the pathogenesis of immune escape that leads to an outbreak of PEDV. Moreover, our study may provide important fundamental information for the future development of novel epitope-based vaccines.

## Methods

### Ethical statement

All procedures involving animals were performed in accordance with guidelines of Institutional Animal Care and Use Committee (IACUC) of National Taiwan University (NTU; Taiwan, Republic of China) and carried out under the regulation and permission of the Institutional Animal Care and Use Committee (IACUC) protocol (No. NTU-103-EL-60) at National Taiwan University (NTU).

### Virus passage and purification

PEDV was isolated and passaged in Vero cells (American Type Culture Collection (ATCC) No. CRL-1586) as previously described^32^. After freezing and thawing, the harvested viral supernatant was centrifuged at 3000 rpm for 30 min to remove cell debris and filtered through a 0.22 µm ultrafiltration cup (Corning, New York, USA). The viral supernatant was then added onto a 20% sucrose (Sigma, Missouri, USA) cushion, purified by ultracentrifugation at 75,000 × *g* for 2.5 h. The viral pellet was re-suspended in phosphate buffered saline (PBS) (Gibco, Gaithersburg, USA), and then applied to a 20–60% sucrose-TNE (20 mM Tris-HCl (pH 7) (Sigma), 100 mM NaCl, 2 mM EDTA (Sigma)) gradient, and centrifuged at 75,000 × *g* for 2.5 h in an Optima™ L-100XP preparative ultracentrifuge using an Avanti J-25 rotor (Beckman Coulter, Sykesville, USA). Purified virions were diluted in TNE buffer, pelleted by centrifugation at 75,000 × *g* for 1.5 h to remove the sucrose and then, re-suspended in TNE buffer.

### mAb production

Three BALB/c mice were intramuscularly (IM) immunized with 20 μg purified PEDV viral particles mixed with 100 μL complete Freund’s adjuvant (Sigma). After two weeks, two IM booster injections were administered using 20 μg purified PEDV viral particles with 100 μL Incomplete Freund’s adjuvant (Sigma) at intervals of 3 weeks. Three days before sacrifice, mice were immunized with 20 μg purified PEDV viral particles in PBS (Gibco) via intrasplenic (IS) injection. Serum antibody titres at each immunization were monitored using a complete PEDV viral particle ELISA and the mouse with the highest titre was sacrificed for hybridoma preparations.

### Hybridoma preparation

Splenocytes were isolated from the mice immunized with purified PEDV particles. After gentle washing with brief centrifugation, splenocytes were fused with SP2 myeloma cells at a cell ratio of approximately 10:1 using 50% polyethylene glycol (Sigma). Hybridomas were seeded onto 96-well culture plates in RPMI-1640 medium supplemented with 20% foetal bovine serum (Gibco), 100 mg/mL streptomycin, and 100 IU/mL penicillin (Sigma), and incubated overnight at 37 °C in a humidified incubator with 5% CO_2_. After incubation, approximately 50% medium was removed from each well, and a selective HAT RPMI-1640 medium (HAT-RPMI) (Sigma) was added to achieve a final concentration of 20% foetal bovine serum (Gibco), 100 mg/mL streptomycin, 100 IU/mL penicillin, 100 mM hypoxanthine (Sigma), 400 mM aminopterin (Sigma), and 16 mM thymidine (Sigma). Wells containing growing hybridoma cells were screened for antibody production by ICC staining using PEDV-infected Vero cells or HEK293 cells (ATCC CRL-1573™) expressing the full-length PEDV S protein. Positive clones were isolated for limiting dilution and incubated in selective HT RPMI-1640 medium without aminopterin. After two limiting dilutions, the supernatant from each line was further tested for anti-PEDV S-specific antibodies by ICC staining using PEDV-infected Vero cells or HEK293 cells expressing the full-length recombinant PEDV S protein.

### mAb purification and quantification

To purify mAbs from cultured supernatants, Pierce™ Protein L Magnetic Beads (Thermo Fisher Scientific, Waltham, USA), which selectively bind to mouse immunoglobulin were utilized following the manufacturer’s instructions. The beads were mixed with 40 mL supernatants of each mAb and incubated for 1 h after which the antibody-bound beads were collected using a magnetic stand. After washing thrice wash buffer (Tris-buffered saline (TBS), 0.05% Tween-20 detergent), the mAbs were eluted with 60 μL elution buffer (0.1 M glycine, pH 2.0) for 10 min and then, alkalized PBS buffer (pH 8.5) was added for neutralization. The concentrations of each purified mAb were determined by the Pierce™ BCA Protein Assay Kit (Thermo Fisher Scientific).

### Construction and expression of various truncated spike proteins

The construction and expression of the full-length PEDV S protein of the Taiwan PEDV-Pintung-52 (PEDV-PT) strain (GenBank: KY929405.1) was performed as previously described^33^. To prepare recombinant truncated PEDV S proteins, regions of the S gene coding for amino acids 1–435 (S^1-435^), 1–485 (S^1-485^), 1–501 (S^1-501^), 1–509 (S^1-509^), 1–575 (S^1-575^), 1–639 (S^1-639^) were amplified using specific primers (Supplementary Table S1 online) and the resultant proteins were purified as described previously^19^. The amplicons encoding different truncated genes were subcloned into a *BamH*I-*Not*I restriction site in the pcDNA^TM^ 3.1/V5-His TOPO^®^ vector (Invitrogen, Carlsbad, CA, USA), transfected into HEK293 cells and selected by using 750 μg/mL Geneticin (G418, Gibco). After two weeks of selection, cells stably expressing the truncated PEDV S proteins were subjected to ICC staining and western blotting.

### Purification and western blot detection of truncated spike proteins

Large-scale purification of the truncated S proteins of PEDV-PT was performed as previously described^19^. The protein expressing HEK-293 cells were harvested, resuspended, and cultured in FreeStyle 293 expression medium (Gibco) for one week. After supernatant collection and removal of cell debris through a 0.22 μm filter, the proteins were banded with HisPur Cobalt Resin (Thermo Fisher Scientific) following the manufacturer’s protocol. The purified proteins were subsequently concentrated with a 30 kDa Vivaspin® 20 (GE Healthcare Life Sciences, NY, USA), cOmplete™ EDTA-free protease inhibitor cocktail (Roche Molecular Biochemicals, Quebec, Canada) was added and the concentrations were measured using the Pierce™ BCA protein assay kit. The purified proteins were denatured in a buffer containing NuPAGE® LDS sample buffer and NuPAGE® reducing agent, and heated for 5 min at 95 °C. Using Bio-Rad Mini-PROTEIN^®^ electrophoresis system(Bio-Rad, Hercules, CA, USA), the samples were separated using 10% sodium dodecyl sulfate (SDS)-polyacrylamide gel electrophoresis (PAGE) cast in a gradient T-Pro EZ Gel Solution (T-Pro Biotechnology, Taiwan) according to the manufacturer’s protocol. Following this, proteins were wet-blotted onto a polyvinylidene difluoride (PVDF) membranes (Bio-Rad)blocked with 5% skim milk in Tris-buffered saline with 0.05% Tween 20 (TBS-T) buffer at room temperature (RT) for 1 h. Membranes were incubated with a 1:5000 diluted anti-V5 tag antibody for 1 h at RT. After washing with TBS-T, horseradish peroxidase (HRP)-conjugated goat anti-mouse IgG secondary antibody (1:10,000 dilution in blocking buffer; Jackson ImmunoResearch Laboratories, Philadelphia, USA) was added and incubated at RT for 1 h, the signals were visualized using Clarity™ Western ECL Blotting Substrates (Bio-Rad) and detected with a ChemiDoc™ XRS+ Imaging System (Bio-Rad).

### Neutralizing assay for the PEDV-PT strain

A neutralizing assay was conducted as previously described with some modifications^32^. Next, 100 μL suspended Vero cells (ATCC No. CRL-1586) were seeded onto 96-well culture plates at 3 × 10^5^ cells/mL and incubated overnight at 37 °C in a humidified incubator with 5% CO_2_ until cells reached 90% confluence. Purified mAbs were two-fold serially diluted from 20 μg/mL to 2.5 μg/mL in post-inoculation medium (PI medium) containing Dulbecco’s modified Eagle medium (Gibco) supplemented with tryptose phosphate broth (0.3%) (Sigma, Missouri, USA), yeast extract (0.02%) (Acumedia, CA, USA), and 10 μg/mL trypsin (Gibco). Fifty microliters of diluted culture supernatant from each hybridoma clone were mixed and incubated with an equal volume of 100 TCID_50_/mL PEDV-PT passage 5 (PEDV-PT-P5) at 37 °C in 5% CO_2_ for 1 h. Then, the mAb-virus mixtures were transferred to the Vero cell monolayer in 96-well tissue culture plates and washed twice with PI medium prior to inoculation. Following incubation at 37 °C for 1 h, inocula were discarded and 100 μL fresh PI medium was added to each well. Plates were incubated at 37 °C for 48 h and cytopathic effects (CPE) were monitored every 24 h.

### PEDV spike-specific mAb selection and epitope mapping by ICC staining

To select PEDV spike-specific mAbs and for epitope mapping of these NmAbs, ICC staining of mAbs to full-length PEDV S and truncated S protein-expressing HEK293 cells were performed. HEK293 cells expressing truncated PEDV spike proteins were seeded onto 96-well culture plates at 80% confluency and fixed using 80% acetone. After air-drying and washing thrice with 200 μL PBS/well, wells were incubated with 200 μL of each purified mAb at 20 μg/mL for 1 h at room temperature. After incubation, wells were washed 3 times with PBS and neutralizing antibodies were detected using a polyclonal anti-rabbit/mouse immunoglobulin EnVision-DAB + system (Dako, CA, USA). After secondary antibody incubation and PBS wash, the signal was detected using 3,3′-diaminobenzidine (DAB) (Dako) according to the manufacturer’s instructions. Reactions were evaluated using an inverted light/fluorescence microscope.

### Epitope mapping by indirect enzyme-linked immunosorbent assay (ELISA)

The procedure of the indirect ELISA has been performed as described previously^33^ with some modifications. Purified S^1-435^, S^1-485^, S^1-501^, S^1-509^, S^1-575^, S^1-639^ and the full-length spike proteins of PEDV-PT were coated onto the Nunc maxi-soap platee (Thermo Fisher Scientific) at 0.034 mM dilution in coating buffer (KPL, SeraCare, MA, USA) at 4 °C for 16 h and were blocked by 300 μL blocking buffer (KPL, SeraCare) at RT for 1 h followed by washing six times with washing buffer (KPL, SeraCare). The purified NmAbs, P4B-1, E10E-1–10, and the non-PEDV recognizing mAb, UNK-1, were serially diluted from 2.5 μg/mL to 78.1 ng/mL and subsequently applied to the plates as primary antibodies. The anti-V5 tag antibody (Invitrogen) was serially diluted from 40,000X to 640,000X and added to the plates as the positive control. Following a 1-h incubation period and washing six times, the 1,000-fold diluted HRP-conjugated goat anti-mouse IgG antibodies (KPL, SeraCare) added to the plate and incubated for 1 h. After washing six times, 50 μL of ABTS® Peroxidase Substrate System (KPL, SeraCare) was used to obtain a positive signal. The coloration procedure was stopped after 20 min by adding 50 μL stopping solution (KPL, SeraCare). The signals were detected at 405 nm wavelength by EMax Plus Microplate Reader (Molecular Devices, Crawley, UK). The final data is shown as a sample to positive ratio (S/P ratio), representing the difference between the OD values of the sample and the negative control divided by the difference between the OD value of the positive and negative controls. The OD value of the positive control was obtained from the result of 80,000X diluted anti-V5 tag antibody, and the OD value of the negative control was obtained from the result of the secondary antibody only wells.

### Linear epitope mapping by immunodot blotting and western blotting

To confirm the conformational significance of the neutralizing epitopes, we also denatured the six purified truncated spike and the full-length spike protein of PEDV-PT by mixing them with NuPAGE® Reducing Agent (Thermo Fisher Scientific) and heating for 5 min at 95 °C. The purified proteins and the denatured proteins were separately dotted on nitrocellulose membranes (Merck Millipore, MA, USA) at 50 ng. The reducing agent mixed with water was also dotted on the membrane as a negative control. Following a 1-h block in 5% skim milk, the membranes were stained with NmAbs (P4B-1 and E10E-1–10) and non-neutralizing mAb (R7H-2) at 2.5 μg/mL diluted in TBS-T buffer at RT for 1 h. After washing three times, HRP-conjugated goat anti-mouse IgG secondary antibody (1:10,000 dilution, Jackson ImmunoResearch Laboratories) was added and incubated at RT for 1 h. Protein signals were visualized using the Clarity™ Western ECL Blotting Substrates (Bio-Rad) and detected with a ChemiDoc™ XRS+ Imaging System (Bio-Rad) as previously described. To ensure the denatured proteins were correctly dotted on the membrane, after the first run of probing, the mAbs were stripped with stripping buffer (Thermo Fisher Scientific) and the membranes were separately re-probed by anti-V5 tag antibodies (Invitrogen).

### Modelling of PEDV S protein

A full-length homology model of the ectodomain of recombinant PEDV S protein was generated by SWISS-Model and built with ProMod3 version 1.0.0 (https://swissmodel.expasy.org). The manipulated protein sequence of the PEDV S ectodomain was constructed using the trimeric human coronavirus NL63 spike structure (PDB accession no. 5SZS) as a template. The images were edited using UCSF ChimeraX molecular modelling system^34^.

## Supplementary information

Nucleotide sequences of the primer sets for the amplification of genes encoding different sized spike (S) proteins

## Data Availability

The datasets generated during and/or analysed during the current study are available from the corresponding author on reasonable request.
