# Supplementary material for: Identification of Neutralizing Monoclonal Antibodies Targeting Novel Conformational Epitopes of the Porcine Epidemic Diarrhoea Virus Spike Protein
Source: Sci Rep. 2019 Feb 21;9:2529. doi: 10.1038/s41598-019-39844-5 (PMC6385244; doi:10.1038/s41598-019-39844-5)
Supplement: Supplementary file 1 — Nucleotide sequences of the primer sets for the amplification of genes encoding different sized spike (S) proteins [file 41598_2019_39844_MOESM1_ESM.docx]

**Identification of Neutralizing Monoclonal Antibodies Targeting Novel Conformational Epitopes of the Porcine Epidemic Diarrhoea Virus Spike Protein**

**Chia-Yu Chang^1^, Ivan-Chen Cheng^2^, Yen-Chen Chang^1^, Pei-Shiue Tsai^2^, Seiu-Yu Lai^2^, Yu-Liang Huang^3^, Chian-Ren Jeng^1,2^, Victor Fei Pang^1,2^, Hui-Wen Chang^1,2*^**

1. Graduate Institute of Molecular and Comparative Pathobiology, School of Veterinary Medicine, National Taiwan University, Taipei 106, Taiwan

2. School of Veterinary Medicine, National Taiwan University, Taipei 106, Taiwan

3. Animal Health Research Institute, Council of Agriculture, New Taipei City 251, Taiwan.

*Corresponding author

Hui-Wen Chang, PhD, DVM

Graduate Institute of Molecular and Comparative Pathobiology,

School of Veterinary Medicine, National Taiwan University,

No. 1, Section 4, Roosevelt Rd., Taipei 10617, Taiwan

E-mail: huiwenchang@ntu.edu.tw

Tel: +886-2-3366-9899

Fax: +886-2-2762-1965

**Supplementary Table S1**

Nucleotide sequences of the primer sets for the amplification of genes encoding different sized spike (S) proteins.

A. The nucleotide sequences of different primer sets. The restriction enzyme recognition sites of *BamHI* or *NotI* in the forward and reverse primers, respectively, are shown with an underline. B. The list of amino acid positions for the different constructs of the truncated S protein in the PEDV-PT strain and its corresponding positions in the PEDV-Brl strain.

A.

| Primer | Primer sequence |
| --- | --- |
| General Forward | 5' AAAACCgAgCTCggATCCAgTACCCTTC 3' (28) |
| Reverse S^1-639^ | 5' AAAAgCggCCgCCggTgACTCCTTCCAg 3' (28) |
| Reverse S^1-575^ | 5' AAAAgCggCCgCCAGTGAAAGGACAGTT 3’ (28) |
| Reverse S^1-509^ | 5' AAAAgCggCCgCCATCATTgAAAgAgggCAgAg 3' (33) |
| Reverse S^1-501^ | 5' AAAAgCggCCgCCgAAAgAgATTggCTgTTCgT 3' (33) |
| Reverse S^1-485^ | 5' AAAAgCggCCgCCATAAAAGCCGTCATC 3' (28) |
| Reverse S^1-435^ | 5' AAAAgCggCCgCCCACGTCATCGTCAGT 3' (28) |

B.

|  | a.a. position in PEDV S (PEDV-PT) | a.a. position in PEDV S (Brl strain) |
| --- | --- | --- |
| S^1-639^ | 1-639 a.a. | 1-642 (1-637) a.a. |
| S^1-575^ | 1-575 a.a. | 1-578(1-573) a.a. |
| S^1-509^ | 1-509 a.a. | 1-512 (1-507) a.a. |
| S^1-501^ | 1-501 a.a. | 1-504 (1-499) a.a. |
| S^1-485^ | 1-485 a.a. | 1-488 (1-483) a.a. |
| S^1-435^ | 1-435 a.a. | 1-438 (1-433) a.a. |
